# The High-Sensitivity C-Reactive Protein to High-Density Lipoprotein Cholesterol Ratio and the Risk of Contrast-Induced Acute Kidney Injury in Patients Undergoing Percutaneous Coronary Intervention

**DOI:** 10.31083/j.rcm2509338

**Published:** 2024-09-23

**Authors:** Linxiao Deng, Hua Chen, Qingbo Xu, Kedong Han, Jin Liu, Shiqun Chen, Jingru Deng, Leigang Tian, Zeliang Li, Xiaozhao Lu, Yong Liu, Yan Liang

**Affiliations:** ^1^The First Clinical School of Medicine, Guangdong Medical University, 524000 Zhanjiang, Guangdong, China; ^2^Department of Cardiology, Maoming People’s Hospital, 525099 Maoming, Guangdong, China; ^3^Department of Cardiology, Guangdong Cardiovascular Institute, Guangdong Provincial People’s Hospital, Guangdong Academy of Medical Sciences, 510080 Guangzhou, Guangdong, China; ^4^Department of Guangdong Provincial Key Laboratory of Coronary Heart Disease Prevention, Guangdong Cardiovascular Institute, Guangdong Provincial People’s Hospital, Guangdong Academy of Medical Sciences, 510080 Guangzhou, Guangdong, China

**Keywords:** contrast-induced acute kidney injury, high-sensitivity C-reactive protein, high-density lipoprotein cholesterol, biological indicators

## Abstract

**Background:**

The high-sensitivity C-reactive protein to high-density lipoprotein cholesterol ratio (CHR) is a novel biomarker associated with coronary artery disease (CAD) risk. This study aimed to analyze the relationship between CHR and contrast-induced acute kidney injury (CI-AKI).

**Methods:**

This retrospective cross-sectional research included 10,917 individuals who underwent PCI. CI-AKI was diagnosed using the Kidney Disease: Improving Global Outcomes (KIDIGO) standard. Univariate and multivariable logistic regression analyses were conducted to examine the association between CHR and CI-AKI, followed by a receiver operating characteristic (ROC) curve of participants to assess the clinical diagnostic performance of CHR on CI-AKI.

**Results:**

A total of 1037 patients (9.50%) developed CI-AKI after PCI. The age of individuals averaged 64.1 ± 11.1 years old, with 2511 females (23.0%). A multivariate logistic regression study revealed that higher CHR levels were linked to higher CI-AKI incidence rates ([Q4 vs. Q1]: odds ratio (OR) = 1.89, 95% confidence interval (CI) [1.42 to 2.54], *p* < 0.001). A restricted cubic spline analysis revealed a linear association between CHR and CI-AKI. ROC analysis indicated that CHR was an excellent predictor of CI-AKI (area under ROC curve = 0.606, 95% CI [0.588 to 0.624]).

**Conclusions:**

A high CHR level is strongly associated with increased CI-AKI incidence, suggesting that CHR may be an independent risk factor for CI-AKI.

**Clinical Trial registration:**

NCT05050877. https://clinicaltrials.gov/study/NCT05050877?tab=results.

## 1. Introduction

After coronary angiography (CAG) or percutaneous coronary intervention (PCI), one of the major complications is contrast-induced acute kidney injury (CI-AKI) [1]. It is now thought to be the third most prevalent cause of hospital-acquired acute kidney injury (AKI), behind drug-induced renal function deterioration and a decrease in renal perfusion [2]. It is related to prolonged hospitalization, increased medical costs, and increased mortality [3, 4], especially in patients undergoing PCI, which has caused a heavy burden on patients’ economies, health, and spirit. Previous studies have suggested that the original factors, such as renal dysfunction (defined as an estimated glomerular filtration rate (eGFR) <60 mL/min/1.73 m^2^), heart failure, coronary heart disease, diabetes, old age, anemia, inflammation, malignant tumors, and ejection fraction can all increase the incidence of CI-AKI [5, 6, 7]. However, the underlying cause of CI-AKI has not been clearly clarified [8], and there is no effective treatment. Therefore, it is crucial to evaluate patients with high-risk characteristics and implement prompt preventative measures to decrease the incidence and dismal prognosis of CI-AKI.

Presently, several risk variables and risk scores have been created for the incidence and outlook of patients with CI-AKI [6, 9, 10], among which Mehran *et al*. [6] proposed a risk score including eight variables to forecast the development of CI-AKI after PCI. However, these prediction models include the type and number of contrast mediums and a full medical history, which is inconvenient in practical application. Recently, the occurrence and prognosis of some inflammatory indicators in CI-AKI patients have also been proven [11, 12, 13, 14]. High-sensitivity C-reactive protein to high-density lipoprotein cholesterol ratio (CHR) is a new laboratory parameter related to inflammation. According to recent research, CHR is a reliable indicator of the presence and severity of coronary artery disease (CAD) and functions as an independent predictor of severe CAD [15]. However, the association between CHR and CI-AKI remains uncertain. Therefore, this retrospective cross-sectional study aimed to investigate the association between CHR and CI-AKI in CAD patients undergoing PCI.

## 2. Materials and Methods

### 2.1 Study Population

This is a multicenter retrospective study with data from the Cardiorenal Improvement II (CIN-II) Study (ClinicalTrials.gov NCT05050877). Patients treated with PCI and/or CAD at five regional central tertiary teaching hospitals in China were enrolled in the CIN-II Study. A total of 29,333 CAD patients who underwent PCI between 2010 and 2020 were included. The following patients met the inclusion criteria: (1) patients over 18 years old receiving PCI treatment; (2) patients who recorded serum creatinine at baseline and within 48 hours of contrast medium exposure. Patients combined with conditions as follows were excluded: (1) hypersensitivity to contrast medium; (2) eGFR <15 mL/min/1.73 m^2^; (3) severe liver and kidney dysfunction or serious infectious diseases; (4) receiving iodine contrast medium within 7 days; (5) administered nephrotoxic drugs within 14 days; (6) possesses a malignant tumor; (7) patients with incomplete clinical data. Finally, 10,917 patients were included in this study. The Guangdong Provincial People’s Hospital Ethics Committee authorized the study, and it adhered to the Helsinki Declaration (No. GDREC2019-555H-2).

### 2.2 Data Collection and Grouping

All patient demographic characteristics and clinical data were obtained from the Hospital Information System, including age, gender, height, weight, complications, past medical history, hematological parameters, medication, and PCI-related information. All hematological parameters were measured by clinical laboratory technicians in the hospital using a standard automatic biochemical analyzer (AU5800, Beckman, USA), and the results were recorded. CHR was calculated using the following formula: CHR = log [hypersensitive C-reactive protein (mg/L)/high-density lipoprotein cholesterol (mmol/L)] [15].

Participants were assigned to four quartiles as follows: Q1 (n = 2730, CHR ≤0.24), Q2 (n = 2729, 0.24 < CHR ≤ 1.41), Q3 (n = 2729, 1.41 < CHR ≤ 2.67), and Q4 (n = 2729, CHR >2.67).

### 2.3 Definitions and Follow-Ups

All patients had baseline measurements recorded at admission and 48 hours following PCI. If the patient had more than one postoperative creatinine measurement within 48 hours after PCI, the highest serum creatinine value was used for the calculation. According to the new CI-AKI diagnostic criteria proposed by the “Kidney Disease: Improving Global Outcomes (KDIGO)” research team, the study was used within 48 hours following the exposure to the contrast media medium, the serum creatinine increased by ≥26.5 µmol/L (0.3 mg/dL) compared with the baseline value, or the serum creatinine increased to ≥1.5 times baseline within the previous 7 days [16].

PCI treatment was performed by experienced interventional cardiologists using standard techniques in accordance with standard clinical practice. Patients undergoing elective surgery were hydrated with a 0.9% sodium chloride solution at a rate of 1 mL/(kg⋅h) 4 hours before and 6–12 hours after surgery and the rate of severe cardiac insufficiency (left ventricular ejection fraction <40% or pulmonary edema) was halved. All clinical drugs, such as antiplatelet aggregation and lipid-lowering, were used according to the patient’s condition.

This study’s main finding was the incidence of CI-AKI, which was determined by comparing serum creatinine levels before and after surgery. All participants were followed up through outpatient visits, hospital records, or telephone interviews.

### 2.4 Statistical Analysis

Data were presented as the mean with standard deviation (SD) or median with interquartile range (IQR) for continuous variables and quantity and frequency (%) for categorical variables. Categorical variables were compared using the Pearson chi-squared test, and continuous variables using the *t*-test. We tested the CHR and CI-AKI relationship using univariate and multivariate logistic regression. The relationship between CHR as a continuous variable and the odds ratio (OR) for CI-AKI was found using restricted cubic splines. To identify the ideal CHR cut-off point and assess the prediction ability of CHR on CI-AKI, receiver operator characteristic (ROC) analysis was performed using the R package “ROCit”. Finally, exploratory analysis was performed among prespecified subgroups. Statistical analyses were conducted using R software (version 4.1.2; R Foundation for Statistical Computing, Vienna, Austria). A two-sided *p*-value < 0.05 indicated the significance of all analyses.

## 3. Results

### 3.1 Baseline Characteristics of CHR Quartiles

The study included 10,917 patients who were undergoing PCI. In the study population, 2511 (23.0%) were female, with an average age of 64.1 ± 11.1 years. A total of 1037 patients (9.50%) developed CI-AKI (Table 1). Patient data were divided into quartiles based on the CHR index. A considerable increase in the incidence rate of CI-AKI accompanied the rise in CHR levels. The incidence rate of CI-AKI was as high as 14.1% in Q4, whereas only 5.8% of patients in Q1 had the condition; comparatively, the incidence of CI-AKI in Q2 (8.4%) and Q3 (9.7%) was significantly higher (*p* for trend < 0.001). Compared with Q1, patients in Q4 were older, had higher levels of neutrophils, white blood cells, fasting plasma glucose, glycosylated hemoglobin, and low-density lipoprotein cholesterol, had a higher incidence of diabetes, CHF, and anemia, and had lower levels of eGFR and left ventricular ejection fraction. Notably, more patients were likelier to use drug diuretics in groups with higher CHR indexes (*p* < 0.001).

**Table 1.** **Baseline Characteristics of CHR Quartiles**.

Variables		Overall (n = 10,917)	CHR ≤0.24	0.24 < CHR ≤ 1.41	1.41 < CHR ≤ 2.67	CHR >2.67	*p*-value
			Q1 (n = 2730)	Q2 (n = 2729)	Q3 (n = 2729)	Q4 (n = 2729)	
Demographic characteristic							
	Age, years	64.1 (11.1)	63.4 (10.7)	64.3 (10.7)	64.0 (11.5)	64.8 (11.6)	<0.001
	Female, n (%)	2511 (23.0)	662 (24.2)	717 (26.3)	599 (21.9)	533 (19.5)	<0.001
	Age >75, years	1787 (16.4)	379 (13.9)	443 (16.2)	445 (16.3)	520 (19.1)	<0.001
	BMI, kg/m^2^	25.4 (17.9)	25.2 (19.1)	26.4 (22.8)	25.9 (17.6)	24.1 (3.5)	0.495
	Systolic BP, mmHg	131.3 (20.5)	133.3 (19.9)	133.5 (19.6)	131.6 (20.4)	127.3 (21.2)	<0.001
	Diastolic BP, mmHg	75.6 (11.1)	75.8 (10.5)	76.2 (11.1)	75.7 (11.0)	74.8 (11.6)	0.001
Medical history							
	Hypertension, n (%)	6225 (57.1)	1490 (54.7)	1588 (58.3)	1640 (60.2)	1507 (55.3)	<0.001
	Diabetes mellitus, n (%)	4100 (37.6)	870 (31.9)	1025 (37.6)	1100 (40.3)	1105 (40.5)	<0.001
	CHF, n (%)	2370 (21.8)	390 (14.3)	465 (17.1)	595 (21.8)	920 (33.8)	<0.001
	Anemia, n (%)	405 (3.7)	73 (2.7)	81 (3.0)	89 (3.3)	162 (5.9)	<0.001
	CI-AKI, n (%)	1037 (9.5)	157 (5.8)	230 (8.4)	265 (9.7)	385 (14.1)	<0.001
Laboratory test							
	SCr, mmol/L	1.1 (1.3)	1.0 (0.6)	1.1 (0.7)	1.2 (0.9)	1.4 (2.3)	<0.001
	eGFR, mL/min/1.73 m^2^	76.4 (28.4)	81.5 (26.0)	78.3 (27.6)	75.3 (29.1)	70.8 (29.8)	<0.001
	TG, mmol/L	1.7 (1.3)	1.6 (1.3)	1.8 (1.6)	1.8 (1.3)	1.5 (1.0)	<0.001
	LDLC, mmol/L	3.0 (1.1)	2.9 (1.2)	3.1 (1.2)	3.1 (1.1)	3.0 (1.0)	<0.001
	HDLC, mmol/L	1.0 (0.3)	1.1 (0.3)	1.1 (0.3)	1.0 (0.3)	0.9 (0.3)	<0.001
	FPG, mmol/L	6.2 (2.2)	5.4 (1.1)	6.2 (2.4)	6.4 (2.3)	6.7 (2.3)	<0.001
	HbA1c, %	6.6 (1.5)	6.4 (1.3)	6.7 (1.5)	6.7 (1.5)	6.8 (1.7)	<0.001
	hs-CRP, mg/L	15.7 (31.1)	0.7 (0.4)	2.6 (1.1)	7.7 (3.4)	51.7 (45.7)	<0.001
	Neutrophils, 10^9^/L	6.1 (3.3)	5.1 (2.8)	5.7 (2.9)	6.0 (3.1)	7.5 (3.6)	<0.001
	WBC, 10^9^/L	9.2 (16.6)	7.8 (3.9)	9.3 (25.8)	9.2 (18.1)	10.4 (9.2)	<0.001
	Lymphocyte, 10^9^/L	1.8 (0.7)	1.8 (0.7)	1.9 (0.8)	1.8 (0.7)	1.7 (0.7)	<0.001
	CHR, mg/mmol	1.5 (1.7)	–0.6 (0.6)	0.8 (0.3)	2.0 (0.4)	3.8 (0.8)	<0.001
	LVEF, %	57.3 (12.2)	60.8 (10.8)	59.0 (11.5)	56.7 (12.2)	52.7 (12.4)	<0.001
Medication							
	Diuretics, n (%)	2552 (24.4)	418 (16.0)	545 (20.7)	657 (25.0)	932 (36.2)	<0.001
	ACEI/ARB, n (%)	7134 (68.3)	1794 (68.7)	1848 (70.0)	1837 (70.0)	1655 (64.3)	<0.001
	CCB, n (%)	1634 (15.6)	434 (16.6)	428 (16.2)	439 (16.7)	333 (12.9)	<0.001
	β-blockers, n (%)	8498 (81.3)	2126 (81.4)	2154 (81.6)	2162 (82.4)	2056 (79.9)	0.142
	Statins, n (%)	10,125 (96.9)	2539 (97.2)	2567 (97.3)	2540 (96.8)	2479 (96.3)	0.169

**Abbreviations**: BMI, body mass index; CHF, congestive heart failure; CI-AKI, contrast-induced acute kidney injury; SCr, serum creatinine; eGFR, estimated glomerular filtration rate; TG, triglyceride; HDLC, high-density lipoprotein cholesterol; LDLC, low-density lipoprotein cholesterol; FPG, fasting plasma glucose; CHR, high-sensitivity C-reactive protein to high-density lipoprotein cholesterol ratio; hs-CRP, high-sensitivity C-reactive protein; WBC, white blood cells; LVEF, left ventricular ejection fraction; ACEI, angiotensin-converting enzyme inhibitor; ARB, angiotensin II receptor blocker; CCB, calcium channel blocker; BP, blood pressure; HbA1c, glycosylated hemoglobin.

### 3.2 The Association between CHR and the Occurrence of CI-AKI

**Fig. 1.**
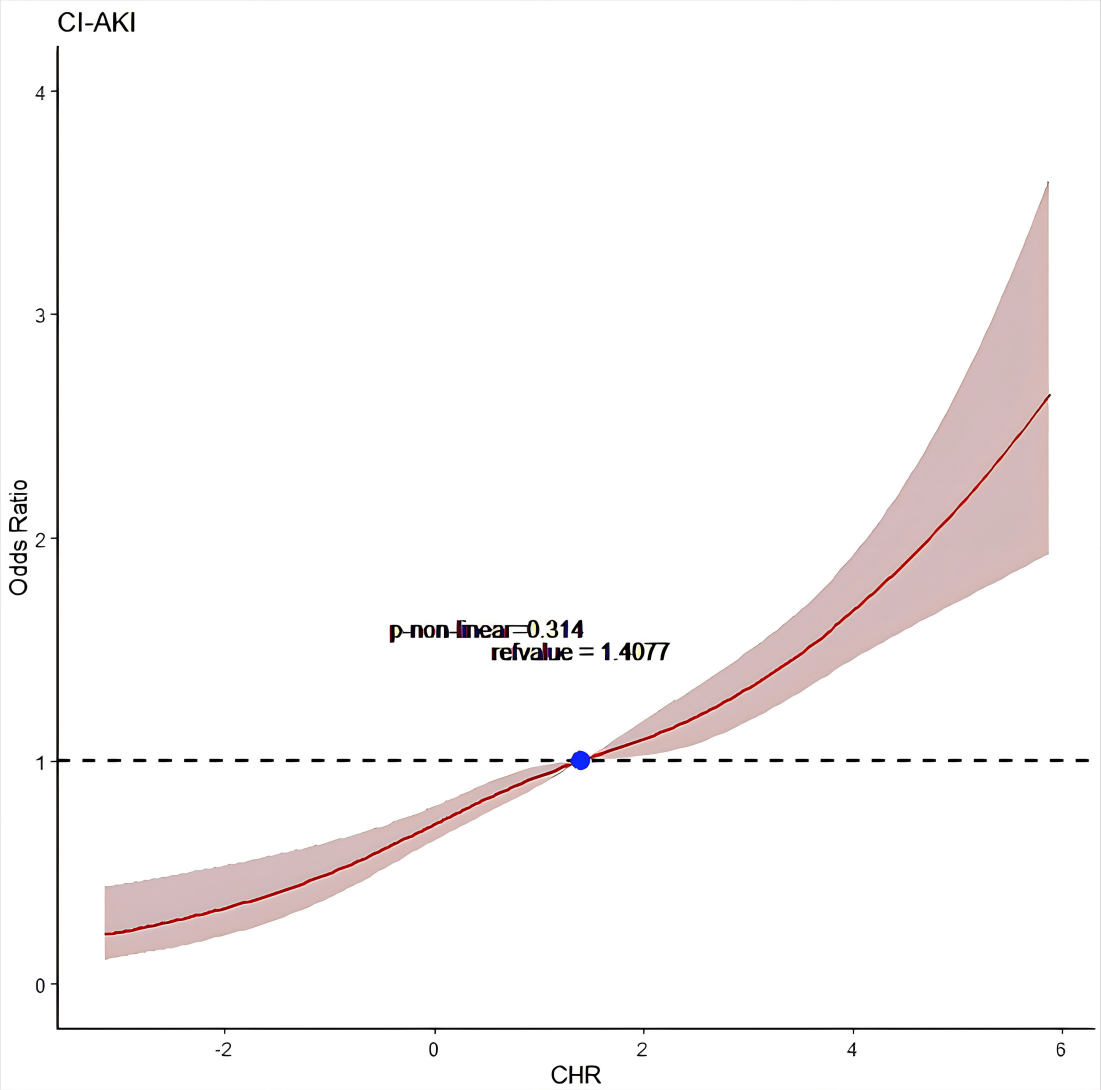
**Restricted cubic spline analysis for exploring the non-linear association between CHR and CI-AKI**. The solid red line shows the adjusted odds ratio of CHR for CI-AKI, and the shaded area around the solid line indicates a 95% confidence interval of the curve. CHR, high-sensitivity C-reactive protein to high-density lipoprotein cholesterol ratio; CI-AKI, contrast-induced acute kidney injury.

We used both univariate and multivariate logistic regression analyses to evaluate the correlation between CHR and CI-AKI risk (Table 2). Model 1 shows that, compared to the reference, the increase in CHR level is connected to the increase in CI-AKI risk, especially in Q2, Q3, and Q4 ([Q2 vs. Q1]: OR = 1.51, 95% CI [1.22 to 1.86], *p* < 0.001; [Q3 vs. Q1]: OR = 1.76, 95% CI [1.43 to 2.17], *p* < 0.001; [Q4 vs. Q1]: OR = 2.69, 95% CI [2.22 to 3.28], *p* < 0.001). Similar results were also shown by Models 2 and 3 (*p* for trends <0.001) following additional and complete adjustments. In addition, a multiple-adjustment restricted cubic spline (RCS) model was used to depict the correlation between the CI-AKI incidence rate and CHR (Fig. 1). These findings suggest that there might be a linear link. With the increase in CHR level, the incidence of CI-AKI increased, which was comparable to the logical regression results (*p* for non-linear = 0.314).

**Table 2.** **The Association between CHR and the Occurrence of CI-AKI**.

	Model 1		Model 2		Model 3	
	OR (95% CI)	*p*-value	OR (95% CI)	*p*-value	OR (95% CI)	*p*-value
CHR						
Q1	1 (Ref)	-	1 (Ref)	-	1 (Ref)	-
Q2	1.51 (1.22–1.86)	<0.001	1.47 (1.19–1.81)	<0.001	1.47 (1.09–2.00)	0.013
Q3	1.76 (1.44–2.17)	<0.001	1.72 (1.40–2.12)	<0.001	2.02 (1.52–2.70)	<0.001
Q4	2.69 (2.22–3.28)	<0.001	2.59 (2.13–3.15)	<0.001	1.89 (1.42–2.54)	<0.001
*p* for trends		<0.001		<0.001		<0.001

**Model 1**: unadjusted logistic proportional odds ratio for contrast-induced acute kidney injury incidence. **Model 2**: logistic proportional odds ratio for contrast-induced acute kidney injury incidence adjusted for age and sex. **Model 3**: logistic proportional odds ratio for the incidence of contrast-induced acute kidney injury adjusted for multiple variables (age, sex, hypertension, congestive heart failure, diabetes, triglyceride, low-density lipoprotein cholesterol, anemia, left ventricular ejection fraction, estimated glomerular filtration rate, angiotensin-converting enzyme inhibitor, angiotensin II receptor blocker, diuretic). CHR, high-sensitivity C-reactive protein to high-density lipoprotein cholesterol ratio; CI-AKI, contrast-induced acute kidney injury.

### 3.3 Analysis of the Predictive Ability of CHR for CI-AKI

**Fig. 2.**
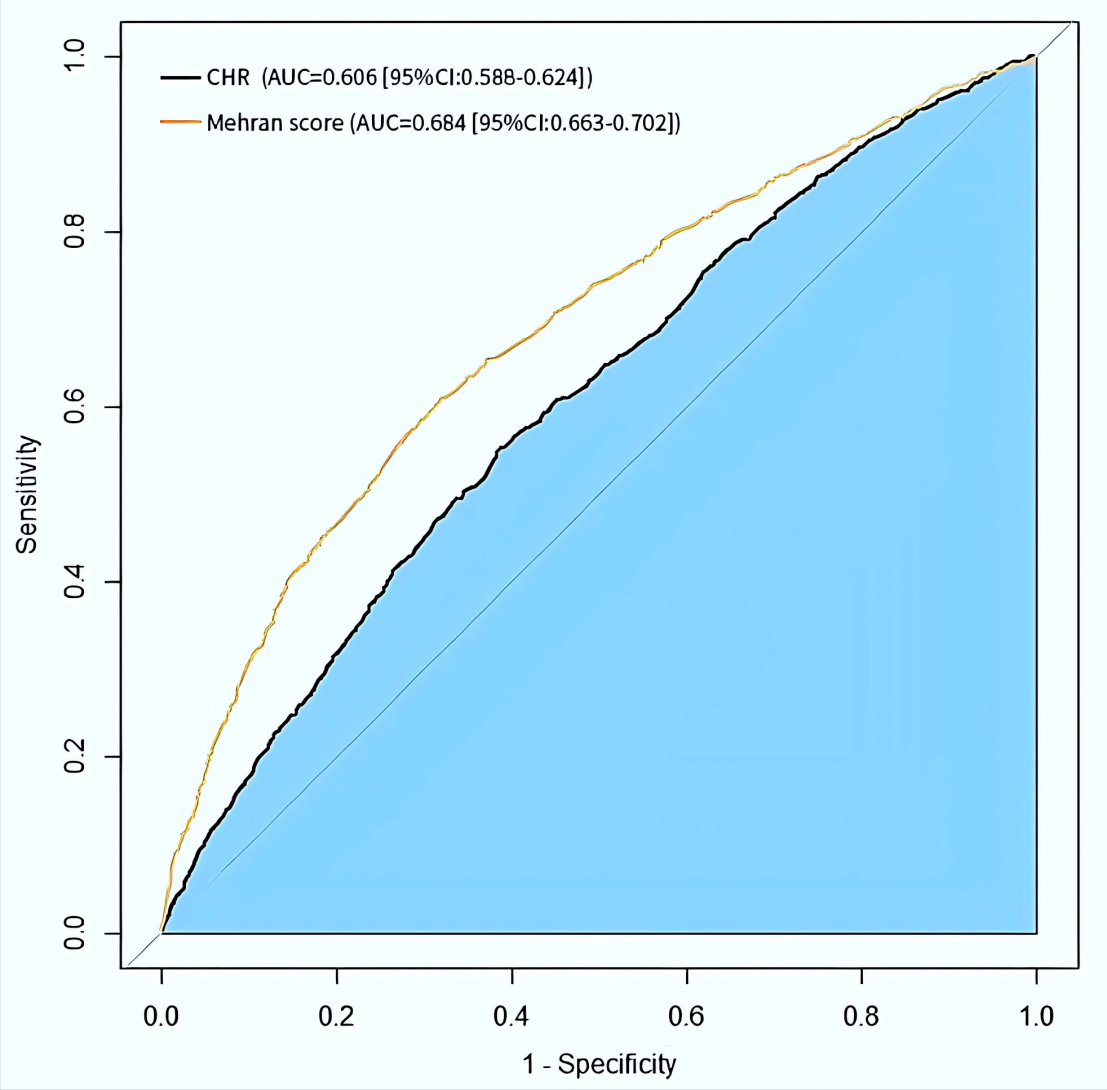
**Receiver operating characteristic curve of CHR and Mehran score for CI-AKI**. CHR, high-sensitivity C-reactive protein to high-density lipoprotein cholesterol ratio; CI-AKI, contrast-induced acute kidney injury; AUC, area under the curve.

Next, the ROC curve was created to assess the Mehran score on the CI-AKI and the clinical diagnostic performance of CHR (Fig. 2). The optimal cut-off value was found to be 1.855, its area under the curve (AUC) was 0.606 (95% CI [0.588 to 0.624]), the corresponding sensitivity was 61.3%, and the specificity was 55.4%, showing satisfactory diagnostic performance. Comparable to the Mehran score for CI-AKI, CHR had a similar predictive value (the AUC of CHR vs. Mehran score was 0.606 vs. 0.684; *p* < 0.001).

### 3.4 Subgroup Analysis of CHR with CI-AKI among PCI Patients

**Fig. 3.**
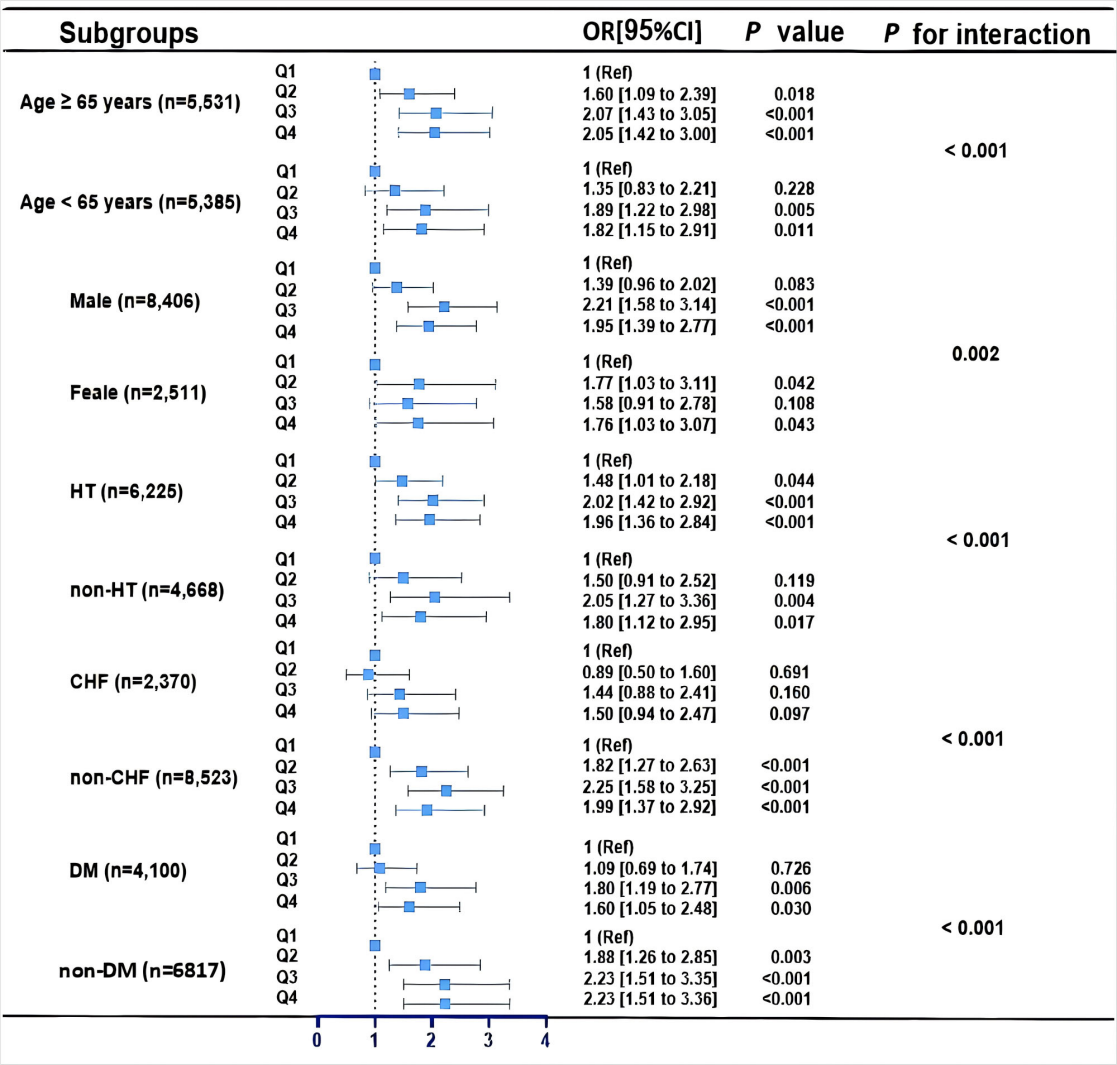
**Subgroup analyses for the association between the CHR and CI-AKI**. Adjusted for age, sex, hypertension, congestive heart failure, diabetes, triglyceride, low-density lipoprotein cholesterol, anemia, left ventricular ejection fraction, estimated glomerular filtration rate, ACEI/ARB. CHR, high-sensitivity C-reactive protein to high-density lipoprotein cholesterol ratio; CI-AKI, contrast-induced acute kidney injury; CHF, congestive heart failure; ACEI, angiotensin-converting enzyme inhibitor; ARB, angiotensin II receptor blocker; HT, hypertension; DM, diabetes mellitus.

Further subgroup analysis was conducted according to age (<65 or ≥65 years), gender (either male or female), hypertension, CHF, and diabetes (Fig. 3). The higher the CHR level, the higher the CI-AKI risk. Most subgroup analysis results are consistent with the overall group results. Notably, CHR is an independent predictor of CI-AKI in the non-CHF subgroup but not in the CHF subgroup. Moreover, the increase in CHR level is related to CI-AKI, whether or not the patient has diabetes.

## 4. Discussion

Within this retrospective study, we mainly discussed the predictive value of the new laboratory parameter CHR on the occurrence of CI-AKI after PCI. The results showed that 1037 occurrences of CI-AKI affected 10,917 patients; the incidence rate was 9.50%, essentially in line with findings from related studies conducted in the US and the UK [17, 18]. Compared to patients with lower baseline CHR levels, those with greater CHR levels had a higher risk of developing CI-AKI. According to the multivariate logistic regression analysis, the CHR level independently predicted the likelihood of CI-AKI patients undergoing PCI. The results of the ROC analysis indicated that CHR had a strong predictive influence on the occurrence of CI-AKI.

Currently, many studies have shown that high-sensitivity C-reactive protein (hs-CRP) or high-density lipoprotein cholesterol (HDL-C) levels have an independent predictive value for CI-AKI. Liu *et al*. [19] prospectively observed 165 patients with STEMI after PCI and found that hs-CRP >16.10 mg/L was an important and independent predictor of CI-AKI. Zhang *et al*. [20] retrospectively studied 1452 STEMI patients undergoing PCI and obtained similar results. The Park *et al*.’s study [21] enrolled 1592 patients receiving PCI treatment from multiple centers and found that low HDL-C levels were linked to a higher chance of long-term mortality and CI-AKI. The latest research shows that CHR is closely related to the occurrence of CAD [15]. However, studies have yet to be conducted on the connection between CHR and CI-AKI. The findings of this study demonstrated that CHR constituted an independent risk factor for CI-AKI in patients undergoing PCI. According to the multivariate logistic regression analysis results, patients with higher CHR levels had a 1.89-fold increased risk of CI-AKI compared to those with lower CHR levels. Therefore, this comprehensive indicator is conducive to identifying high-risk groups, taking advanced preventive measures, and preventing the occurrence of CI-AKI through individual and precise treatments.

The pathophysiological mechanism of CI-AKI is complex [8] and has yet to be clearly clarified. The mainstream theory is the direct and indirect effects of contrast agents on renal function and the disorder of hemodynamics [22]. In its direct mechanism, contrast medium has a toxic effect on renal tubular epithelial cells, leading to loss of function, apoptosis, and necrosis. The indirect mechanism is related to vascular endothelial cells, nitric oxide, prostaglandins, and other vasoactive substances that mediate vascular injury. In addition, the relatively low oxygen partial pressure of the extrarenal medulla, coupled with the increased metabolic demand, makes medullary abnormalities vulnerable to the hemodynamic impact of the contrast medium [22]. Current studies have shown that inflammatory factors play a central role in CI-AKI occurrence [23]. Elevations in hs-CRP are associated with endothelial dysfunction, resulting in vascular injury, reduced blood flow to the kidneys, and reduced renal function [24]. Concurrently, the increase in hs-CRP level is related to the decrease in nitric oxide production, which is also the reason for CI-AKI. On the contrary, HDL-C has a protective effect on atherosclerosis, which has antioxidants, is anti-inflammatory, and can reverse the transit of cholesterol [25]. Theoretically, the increase in hs-CRP count and the decrease in HDL-C level may further accelerate atherosclerosis and raise the chance of unfavorable cardiovascular events. The correlation between CHR and CAD has recently been shown [15]; however, the relationship with CI-AKI has yet to be reported. In this study, we showed for the first time that high CHR levels are a good predictor of CI-AKI after primary PCI.

The occurrence of CI-AKI is closely related to some chronic basic diseases and the age of the patient. At present, studies conducted both domestically and internationally indicate that the following conditions increase the risk of CI-AKI: Renal insufficiency, diabetes, congestive heart failure, old age (>75 years old), contrast medium type and dosage, and other factors. Old age (>75 years old) is an independent risk factor for CI-AKI, which may be related to the fact that older patients are often accompanied by chronic diseases, such as hypertension, diabetes, and CAD, which are likely to lead to renal ischemia, thereby causing the decline in the glomerular filtration rate. Marenzi *et al*. [26] found that among 208 acute myocardial infarction (AMI) patients undergoing primary PCI, the incidence of CI-AKI in older patients (>75 years old) reached 36%. In a study of 8357 PCI patients, Mehran *et al*. [18] found that the incidence of CI-AKI was 21.8% in older patients (≥75 years old). According to this study, the rate of CI-AKI in older PCI patients reached 29.3%, which is similar to previous results. Therefore, in some high-risk patients with chronic diseases, it is significant to perform risk stratification and early intervention through some simple and rapid identification of CI-AKI biomarkers.

Presently, there is no precise or reliable way to treat CI-AKI, although there are many studies on the effectiveness of preventing CI-AKI [2, 27, 28], including capacity expansion with isotonic saline or sodium bicarbonate before PCI, anti-oxidation treatment with N-acetylcysteine or ascorbic acid [29], and the use of low osmolality or isotonic contrast medium [28]. In addition, many studies have demonstrated that statins can lower CI-AKI incidence [30, 31, 32], mostly by enhancing endothelial function, preserving nitric oxide generation, and reducing platelet adhesion, inflammation, and oxidative stress. Statins can lower the risk of renal failure and CI-AKI in individuals with CAD, even if they are administered before PCI. However, not all patients can start these preventive treatments in time. Previous research by Moroni *et al*. [33] has demonstrated a reduced risk of CI-AKI when intravenous infusion is started before surgery. However, patients with heart failure symptoms may be unable to replenish water fully. According to this study, patients with congestive heart failure had a much higher incidence of CI-AKI, up to 45.4%, meaning it is important to identify this high-risk group in time. It is worth noting that our further subgroup analysis shows that, for the congestive heart failure patient population, the predictive indicator of CHR is not significantly related, which may be caused by the high inflammatory indicators of congestive heart failure patients themselves. The specific situation needs further exploration.

## 5. Limitation

This study also had some limitations. First, this study only detected the baseline concentration of hs-CRP before surgery and did not monitor the changes in inflammatory biomarkers during the study. Second, this study only included some commonly used inflammatory markers; other inflammatory indicators, such as interleukin-1, tumor necrosis factor, and neutrophil gelatinase-associated lipocalin, were not routinely detected in this center; thus, a comparative analysis could not be performed. Finally, many patients who did not recheck the blood serum creatinine value within 48 hours after surgery were excluded from the screening, which may cause selection bias due to the lack of data.

## 6. Conclusions

In conclusion, our study shows that among CAD patients undergoing PCI, patients with high CHR levels have a higher CI-AKI probability than those with low CHR levels. CHR has potential predictive value for CI-AKI and may play an independent role in risk stratification in clinical practice.

## Availability of Data and Materials

The datasets used and/or analyzed during the current study are available from the corresponding author on reasonable request.
